# Reflectance confocal findings in a large-cell acanthoma with histologic correlation

**DOI:** 10.1016/j.jdcr.2021.08.016

**Published:** 2021-08-21

**Authors:** Alli J. Blumstein, Katharine L. Hanlon, Wei-Shen Chen, George Elgart, James M. Grichnik, Lilia Correa-Selm

**Affiliations:** a Department of Dermatology, Northwestern University, Feinberg School of Medicine, Chicago, Illinois; b Department of Cutaneous Oncology, Scully Welsh Cancer Center, Cleveland Clinic Indian River Hospital, Vero Beach, Florida; c Department of Dermatology and Cutaneous Surgery, University of South Florida, Tampa, Florida; d Department of Dermatology and Cutaneous Surgery, Dermatopathology Laboratory, University of Miami Miller School of Medicine, Miami, Florida

**Keywords:** confocal microscopy, dermoscopy, histology correlation, large-cell acanthoma, lentigo maligna, lentigo, LCA, large-cell acanthoma, RCM, reflectance confocal microscope

## Clinical presentation

**Fig 1:**
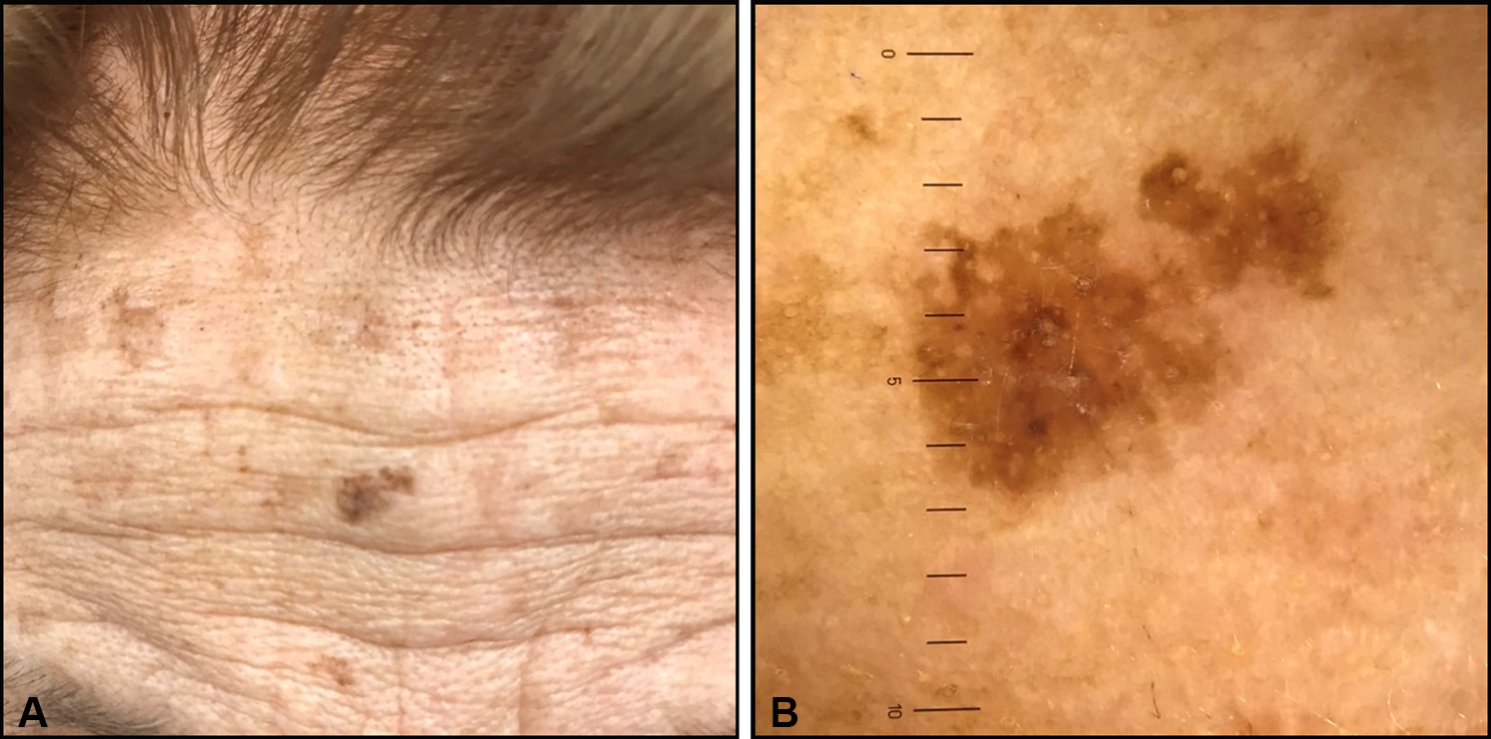
**A**, Clinical photograph and (**B**) dermoscopic image of large-cell acanthoma showing nonuniform shape with equivocal dermoscopic characteristics.

The patient, a 91-year-old woman with no history of skin cancer, presented with an 8-mm flat, irregularly pigmented macule on the forehead for 2 years (Fig 1, *A*). The lesion had been asymptomatic, and the patient was not aware of the changes in its appearance. Contact nonpolarized dermoscopy showed an irregularly shaped lesion with homogeneous pigmentation and focal darker pigmentation. Occasional circles within circles and perifollicular pigmentation were noted (Fig 1, *B*).

## Confocal microscopy appearance

**Fig 2:**
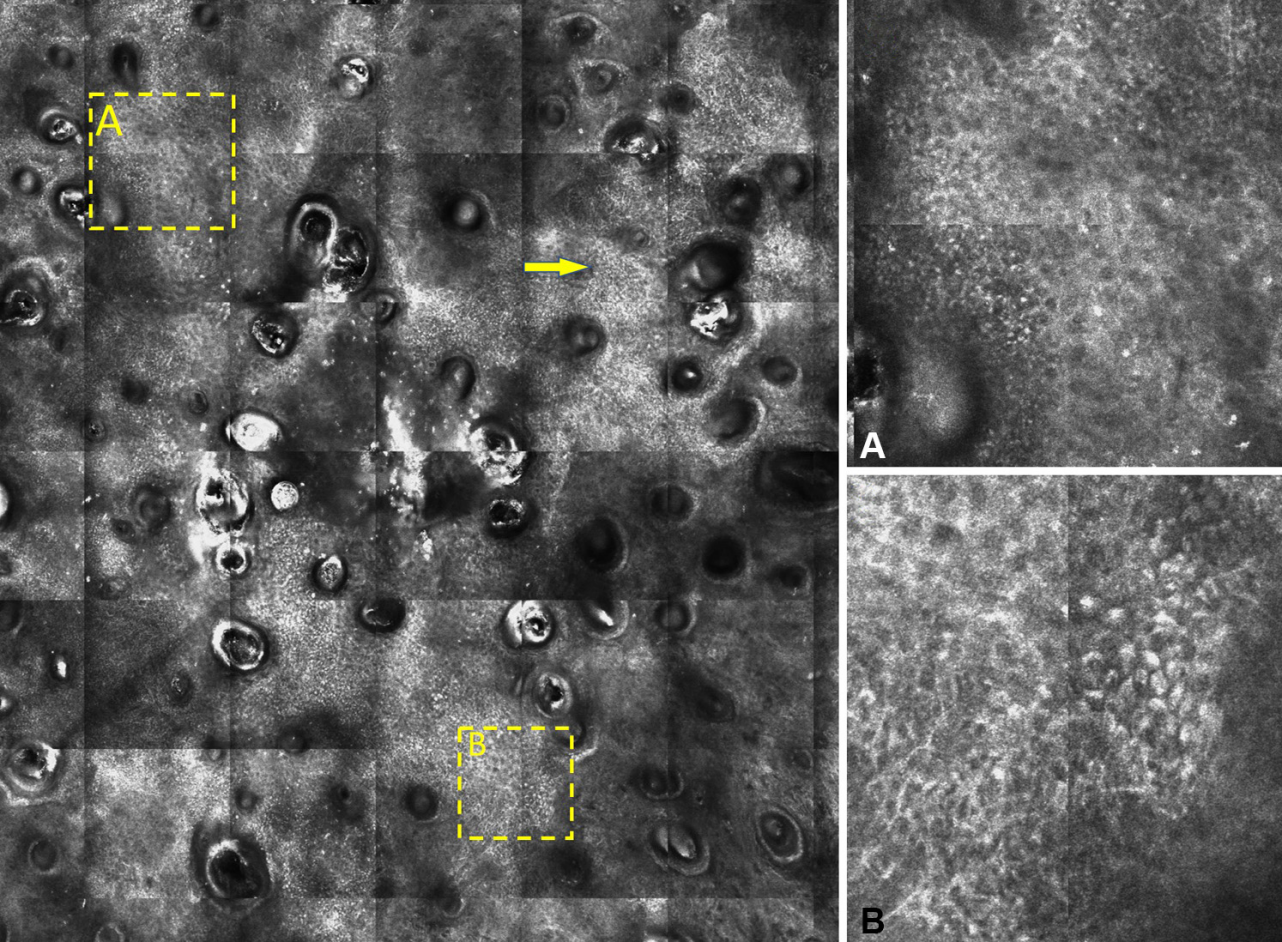
Reflectance confocal microscopy of large-cell acanthoma (at the low-power view) showing a broadened honeycomb pattern (**A**), nonedged and partially edged dermal papillae and highly refractile dendritic cells in sheets (*arrow*), and atypical cobblestone pattern with cells of different sizes and visible nuclei (**B**).

The lesion was examined using the VivaScope 1500 (Caliber Imaging and Diagnostics) reflectance confocal microscope (RCM). In the epidermis, confocal microscopy revealed a broadened, irregular honeycomb pattern with ill-defined borders (Fig 2, *A*). It was interrupted by an atypical cobblestone pattern, consistent with cell size variation and enlarged nuclei within the epidermis (Fig 2, *B*). Strikingly, sheets of highly refractile cells with dendritic extensions were noted toward the base of the epidermis, above the dermoepidermal junction. Within the papillary dermis, small, ill-defined refractive cells with morphologic features consistent with inflammatory cells were also present (data not shown). Given the presence of sheets of dendritic cells under RCM, a shave biopsy was performed and submitted for histologic examination to rule out melanoma in situ.

## Histologic diagnosis

**Fig 3:**
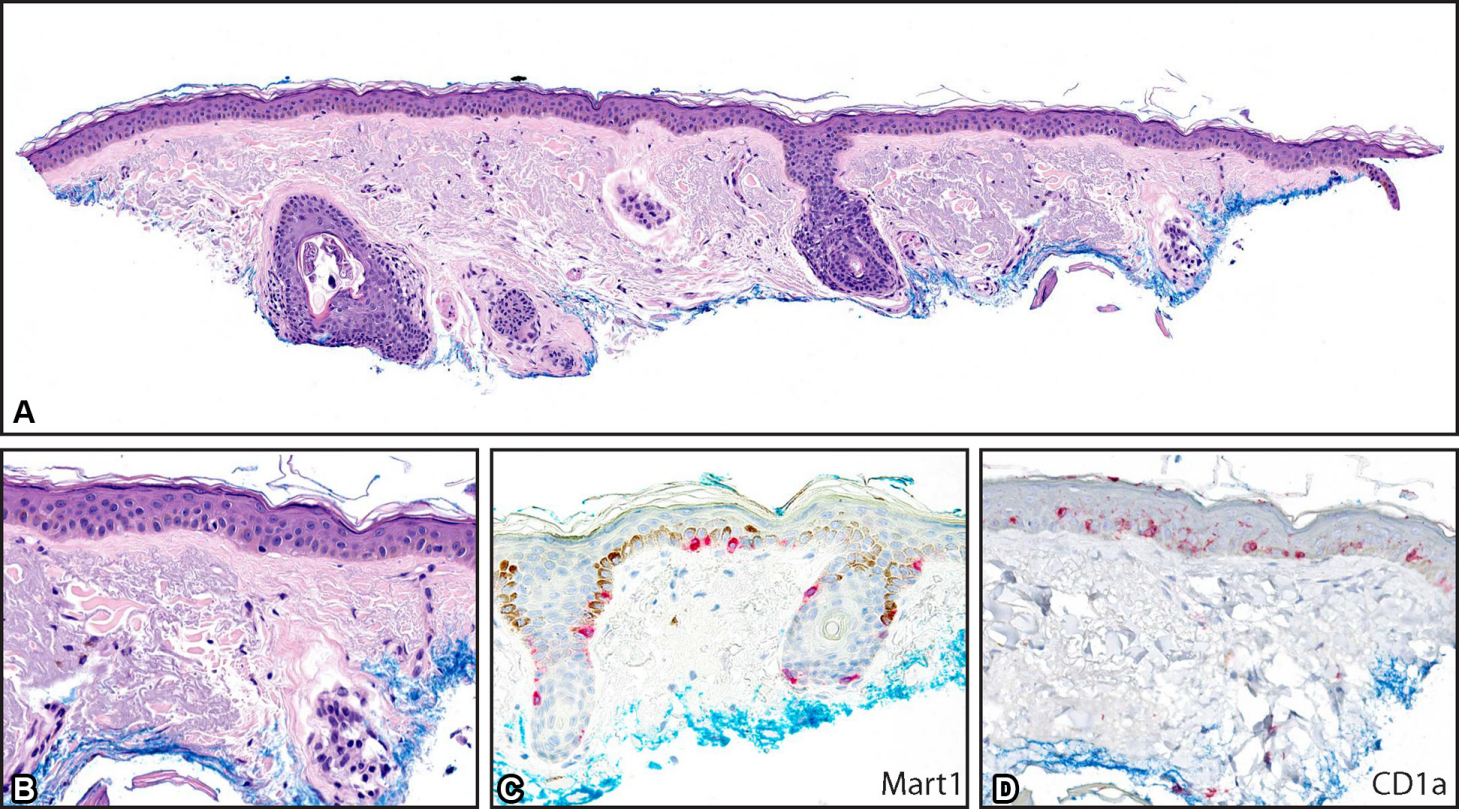
**A**, Histopathology of large-cell acanthoma, at low power, illustrates the solar elastosis and a relatively flattened junction. **B**, The hematoxylin-eosin–stained section, at high power, shows enlarged nuclei of keratinocytes, corresponding to the cellular findings of reflectance confocal microscopy. **C**, At high power, Mart1 stain shows nonconfluent, basally oriented junctional melanocytes. **D**, CD1a immunostaining reveals abundant intraepidermal Langerhans cells. (**A** and **B**, Hematoxylin-eosin stain; **C**, Mart1 stain; **D**, CD1a stain.)

Examination of hematoxylin-eosin–stained sections showed epidermis with enlarged nuclei with basal hypermelanosis (Fig 3, *A* and *B*). A Mart1 immunostain highlighted scattered junctional melanocytes, without significant confluence, nesting, or upward pagetoid migration (Fig 3, *C*). Ki-67 immunostaining stained the keratinocytes and may have shown minimally increased cell proliferation in areas of increased melanocytes (data not shown). Solar/actinic elastosis was noted throughout the dermis. Taken together, a diagnosis of large-cell acanthoma (LCA) was rendered.

CD1a immunostaining was performed and also revealed dendritic Langerhans cells within the epidermis (Fig 3, *D*).

## Discussion

RCM has proved its clinical utility as a noninvasive tool to aid in the diagnosis of benign and malignant skin lesions in real time.^1^ Specific cell morphology and architectural configuration at various levels of the skin have been identified, which accurately delineate pathologies; however, some lesions still present challenges. The nonedged and partially edged dermal papillae and dendritic melanocytes in sheets and the nucleated cells were concerning for lentigo maligna,^1^ whereas the broadened, irregular honeycomb pattern in the epidermis with cells of various sizes raised the possibility of an actinic keratosis^2^ or squamous cell carcinoma in situ. These findings necessitated a biopsy to differentiate the diagnoses.

Based on the enlarged keratinocytes and lack of significant cytologic atypia under light microscopy, a diagnosis of LCA was rendered. Despite the impression of increased dendritic cells under RCM, the Mart1 immunostain unexpectedly revealed considerably fewer melanocytes at the junction in this lesion.

Activated Langerhans cells are reported to appear as the brightly reflecting dendritic cells under RCM and may pose as morphologic mimickers to melanocytes, leading to diagnostic challenges for pigmented lesions in RCM.^3^ Our CD1a immunostain indeed highlighted abundant intraepidermal Langerhans cells that may have contributed to the apparent dendrite sheets.

In this case, pigmented keratinocytes comprise the bulk of the cells. Their confocal appearance as nucleated cells correlates with the histologic morphology of large cells in the epidermis. It is likely that the ultraviolet-damaged keratinocytes create the cytokine environment favorable for melanocyte stimulation and/or Langerhans cell activation. Of course, neoantigens generated by ultraviolet mutations could also lead to Langerhans cell activation.

There is another case report of LCA on RCM, which showed findings similar to squamous cell carcinoma.^3^ There has been deliberation in medical literature for decades about whether LCAs are their own class of benign neoplasms or a variant of other lesions such as solar lentigo, actinic keratoses, seborrheic keratoses, or Bowen disease.^4^, ^5^, ^6^ Future studies will help discern the interactions between these cell types and discriminate biologic potential.

## Conflicts of interest

Dr Grichnik serves as a consultant for Galileo Group, Canfield Scientific and has previously received equipment and meeting support from Caliber Imaging and Diagnostics. Dr Correa-Selm served as speaker of Accutec Blades and was a consultant for Castle Biosciences. Authors Blumstein and Hanlon and Drs Chen and Elgart have no conflicts of interest to declare.
